# Is health literacy related to health behaviors and cell phone usage patterns among the text4baby target population?

**DOI:** 10.1186/2049-3258-72-13

**Published:** 2014-05-07

**Authors:** Elisabeth Poorman, Julie Gazmararian, Lisa Elon, Ruth Parker

**Affiliations:** 1 Cambridge Health Alliance, 1493 Cambridge Street, Cambridge, MA, USA; 2 Department of Epidemiology, Rollins School of Public Health, Emory University, 1518 Clifton RdNE, Atlanta, Georgia; 3 Department of Biostatistics, Rollins School of Public Health, Emory University, 1518 Clifton RdNE, Atlanta, Georgia; 4 School of Medicine, Emory University, 1648 Pierce DrNE, Atlanta, Georgia

**Keywords:** Health literacy, Health promotion, Maternal and child health, mHealth, Postnatal, Prenatal, Text messaging, Underserved populations

## Abstract

**Background:**

Text4baby provides educational text messages to pregnant and postpartum women and targets underserved women. The primary purpose of this study is to examine the health behaviors and cell phone usage patterns of a text4baby target population and the associations with health literacy.

**Methods:**

Pregnant and postpartum women were recruited from two Women, Infant and Children clinics in Atlanta. Women were asked about their demographics, selected pregnancy or postpartum health behaviors, and cell phone usage patterns. Health literacy skills were measured with the English version of the Newest Vital Sign. Multivariable logistic regression was used to examine health behaviors and cell usage patterns by health literacy classification, controlling for commonly accepted confounders.

**Results:**

Four hundred sixty-eight women were recruited, and 445 completed the Newest Vital Sign. Of these, 22% had inadequate health literacy, 50% had intermediate health literacy, and 28% had adequate health literacy skills. Compared to adequate health literacy, limited literacy was independently associated with not taking a daily vitamin during pregnancy (OR 3.6, 95% CI: 1.6, 8.5) and never breastfeeding their infant (OR 1.4, 95% CI: 1.1, 1.8). The majority (69.4%) of respondents received nine or more text messages a day prior to enrollment, one in four participants (24.6%) had changed their number within the last six months, and 7.0% of study participants shared a cell phone. Controlling for potentially confounding factors, those with limited health literacy were more likely to share a cell phone than those with adequate health literacy (OR 2.57, 95% CI: 1.79, 3.69).

**Conclusions:**

Text4baby messages should be appropriate for low health literacy levels, especially as this population may have higher prevalence of targeted unhealthy behaviors. Text4baby and other mhealth programs targetting low health literacy populations should also be aware of the different ways that these populations use their cell phones, including: sharing cell phones, which may mean participants will not receive messages or have special privacy concerns; frequently changing cell phone numbers which could lead to higher drop-off rates; and the penetrance of text messages in a population that receives many messages daily.

## Background

Poor pregnancy outcomes, including low birth weight and preterm birth, continue to be a problem in the US, particularly for minority women and those with few resources [1]. Rates for low birth weight and preterm births have remained relatively constant since 1980, with improvements in infant mortality attributable primarily to advanced health care interventions for preterm infants as opposed to increased utilization of preventive services. Infant mortality remains high in the United States when compared with other industrialized countries, with a rate of 6.42/1,000 live births between 2008 and 2009 [2].

Unhealthy behaviors in the prenatal period, including smoking, alcohol use, and poor diet, are linked to poor pregnancy outcomes [1]. Conversely, proactive healthy behaviors in the preconception and prenatal period, such as vitamin use, influenza vaccine, and regular prenatal care lead to improved outcomes [1,3]. While behavior modification has had limited success in modifying poor pregnancy outcomes, the combined effect of a multi-pronged behavior intervention has the possibility to have a significant impact on poor pregnancy outcomes [4]. Ideally, these interventions would be targeted to women who have the greatest potential to benefit. Women at higher risk of poor outcomes, however, have traditionally been the most difficult to reach.

The recent explosion of new technologies offers novel opportunities for counseling and behavior change for these historically underserved groups. Text messaging is unique among newer technologies as it is widely used across income and educational strata. According to the Pew Research Center’s Global Attitudes Project, a survey of 21 representative countries found that 85% of those surveyed owned a cell phone, and of those, 75% reported regularly using text messaging [5]. Significantly, text messaging was more common in the poorest nations surveyed. In a separate Pew Research Center survey of Americans conducted in 2011, 83% of Americans owned a cell phone, and 73% of cell phone owners used text messaging [6]. The groups who sent the most text messages were young (18–24 years), earned less than $30,000 a year, and had less than a high school education.

Text messaging is thus a potentially powerful avenue for reaching low-resource populations, and has led to the creation of mobile health interventions, known as mhealth programs. One of the few programs that focuses on maternal and infant health, text4baby, sends educational messages to pregnant and postpartum women with the goal of promoting healthy, preventative behaviors. The program was created by a public-private partnership overseen by the National Healthy Mothers, Healthy Babies Coalition. Text4baby developed a series of messages from evidence-based guidelines from the American College of Obstetrics and Gynecology and the Bright Futures Guidelines for Infants, Children, and Adolescents. Participants in the text4baby program receive one free educational message three times a week timed to their gestational age or infant’s birthdate.

Text4baby’s educational messages were refined in focus groups at community centers in six cities across the country and are aimed at women with low health literacy, with messages written at a sixth grade level [7]. This target population therefore likely overlaps with the group most likely to text: young, less educated, and low-income women. Defined as “the degree to which individuals can obtain, process, and understand basic health information and services needed to make appropriate health decisions”, health literacy has emerged as a marker of existing knowledge, the ability to process new health information, and a strong predictor of health behaviors [8]. Some studies have indicated that it is a stronger predictor of outcomes than education alone [9]. Targeting women with low health literacy for health education could potentially have the greatest impact, encouraging women at high risk for poor pregnancy outcomes to make healthy decisions for themselves and their children [10].

The effect of health literacy on outcomes may be mediated by higher rates of unhealthy behaviors. A 2011 meta-analysis conducted for the Agency for Healthcare Research and Quality found that low health literacy is associated with lower acceptance of influenza vaccine and decreased ability to interpret health messages [11]. Other studies have found that women with low health literacy are less likely to breastfeed [12], plan their pregnancy [13], and to be insured [14]. These associations are not consistent across all studies, suggesting that other factors, such as attitudes and cultural beliefs about medicine, may mediate the effect of health literacy on health behaviors [15]. More information is needed about how the prevalence of these behaviors varies with health literacy and how to best support behavior change in these populations.

In addition to differences in health behaviors, successful mhealth education requires intimate knowledge of the way that target populations use their cell phones. Though women who are likely to have low health literacy have adopted text messaging, they may use it in different ways. Younger cell phone users in the US are more likely to share a cell phone, for instance, and lower income cell phone users are more likely to use prepaid cell phone plans [6]. Those with prepaid plans are in turn less likely to use text messaging, and to change their numbers frequently. Americans with higher education are more likely to look for health information on their phones, as are ethnic minorities [16]. Since text message is a written medium, health literacy may influence the type of messages that users send and receive, and their understanding and use of these messages.

We explored the prevalence of healthy behaviors in a group of pregnant and postpartum women enrolled in text4baby, and their relationship with health literacy skills. In addition, we explored the relationship between cell phone usage characteristics and health literacy skills to better understand how mhealth programs like text4baby can potentially be used to improve maternal and child health in high risk populations.

## Methods

### Study design

Cross-sectional survey using a stratified random sample design.

### Setting

The study was conducted in two Special Supplemental Nutrition Program for Women, Infants, and Children (WIC) Clinics in Metro Atlanta as part of a broader evaluation of text4baby in this population.

### Study population

Women were recruited from nutrition classes (which are mandated for all those receiving WIC support) at the two WIC clinics. Interviewers attended all classes during the study collection period, and either approached all women in the class if the class was small, or randomly selected participants using numbered slips of paper, creating a stratified random sample. Women who were willing to participate were eligible for the study if they: 1) were the biological mother of a child under 10 months old (postpartum) or were currently pregnant; 2) had a working cell phone; 3) could receive text messages; 4) had not been enrolled in text4baby previously; and 5) spoke English. Those who qualified to participate were consented orally with both the Emory University approved consent and HIPAA agreement forms. Recruitment procedures have been described previously in more detail [17].

### Data collection

Participants were read an in-person pregnant or postpartum baseline survey by a trained interviewer to ensure comprehension, which took approximately ten to fifteen minutes. Data was collected at three points: baseline, two weeks, and two-to-six month follow-up. This paper analyzes baseline data.

### Measures

At the baseline interview, women self-reported all demographic, behavioral, and cell usage data. Women were asked about behaviors that depended on whether they were pregnant or postnatal: 1) All women were asked if they currently smoked (possible answers “no”, “some days”, or “every day”); if they had rules about smoking in the house (“no”, “no one is allowed to smoke in the house”, or “people are allowed to smoke in some rooms sometimes”); how often they felt “down-hearted or blue”, (“all of the time”, “most of the time”, “some of the time”, “a little of the time”, “none of the time”); if they had had an alcoholic drink in the past thirty days (“yes”, “no”, or “don’t know”). 2) Pregnant women were asked how many days a week they participated in physical activity for thirty minutes or more (“less then one day a week”, “one to two days”, “three to four days”, or “five a more”, or that they were advised against exercise by a health professional”); if they had a seasonal flu shot in the last year (“yes”, “no”, “don’t know”); how often they kept their appointments (“always”, “nearly always”, “sometimes”, “seldom”, and “never”); and how often they took a multivitamin in the past week (“I did not take any vitamins at all”; “1-3 times a week”, “4-6 times a week”, “daily”). 3) Postpartum participants were asked if they were currently breastfeeding, and if not, if they breastfed at any point after birth; and how often they put their baby in a car seat (“always”, “nearly always”, “sometimes”, “never” and “don’t have a car”). All answers were collapsed into healthy and unhealthy behaviors; for instance, those who smoked sometimes or always versus those who did not smoke. These collapsed categories are presented in the results.

The other outcomes were cell phone usage patterns. Women were asked the average number of text messages they received per day (“less than 2 per day;” “3 to 5 per day;” “6 to 8 per day;” “9 or more per day”). Answers were recoded into “9 or more per day” versus all others. Women were also asked if they currently shared a cell phone (“yes” or “no”). Finally, women were asked how many phone numbers they had had in the past six months (“1, 2, 3, 4, 5, >6”). They were classified into more than one versus one cell phone number.

The primary predictor was health literacy. This was measured during the final portion of the baseline survey using the Newest Vital Sign (NVS) assessment [18]. The NVS is a six-question instrument that asks respondents to interpret an ice cream label, and incorporates both reading literacy and numeracy skills. In the original paper on this health literacy metric, the creators of the NVS found that those with a score less than two on the English version were likely to have inadequate health literacy, and those with a score of four or greater were likely to have adequate health literacy when measured against the Test of Functional Health Literacy in Adults. Women were divided into three health literacy categories: 0–1 for limited health literacy, 2–3 for intermediate health literacy, and 4–6 for adequate health literacy [18].

Demographics were included as covariates: education level (less than high school, high school or GED, or beyond high school), ethnicity (black versus all others), income (less than $10,000, $10,001 to $20,000, and more than $20,000), employment (any current employment versus all others), and marital status (living with a partner/married versus all others).

### Data analysis

All analyses took into account the sampling design and weights (based on selection probability, adjusted for non-response) using SAS v9.3 survey procedures (SAS Institute, Cary, NC, USA) so that reported estimates reflect the clinic population. Demographics, behaviors, and cell phone usage characteristics were summarized by median and interquartile range or proportions, overall and by NVS. The statistical significance of crude associations between NVS and other characteristics are evaluated with the Rao-Scott likelihood ratio chi-square test. Multiple logistic regression with a generalized logit function was used to evaluate the association of health literacy categories and those health behaviors that were significantly associated in crude tests, controlling for common confounders income and education (p = .05). The association of health literacy and cell phone usage characteristics were similarly assessed, controlling for the most common confounders identified in the literature: age, education, income, employment status, and marital status [11]. Race was not controlled, as our sample was more than 90% African American. Linearity of the logit for age was confirmed and multicollinearity and other model diagnostics were performed. The type I error rate (alpha) was set at 0.05.

## Results

### Baseline characteristics

A baseline survey was read to 468 women, and the 445 participants who completed the NVS were included in the analysis. Participants had an estimated median age of 25 (Table 1). The majority (92.3%) of study participants were African American; 57.3% had twelve or fewer years of schooling, and 81.1% had a household income under $20,000. Slightly more than half (56.7%) of participants were unemployed or students, and 29.7% were married or living with a partner. Health literacy scores overall were low, with 22% having limited health literacy, 50% intermediate health literacy, and 28% adequate health literacy. Higher health literacy was significantly associated with older age, higher education, higher income, and being employed. Race and marital status and racial distribution did not differ significantly between health literacy categories.

**Table 1:** Demographics stratified by newest vital sign, text4baby programs, two metro Atlanta WIC clinics, 2012

	**Overall n = 445**		**Newest vital sign score**			
			**Limited 0-1**	**Intermediate 2-3**	**Adequate 4-6**	
			n = 93, (22%)*	n = 226, (50%)*	n = 126, (28%)*	
	**n**	Median* and IQR**				**p-value**^**+**^
**Age, years**	438	25.0 (21–30)	25.0 (21–31)	27.0 (22–31)	27.0 (22–31)	<0.01
		n (percent*)				<0.01
**Race/ethnicity**	444					0.05
Black, Non-Hispanic	410	92.3%	84 (90.3%)	213 (94.3%)	113 (90.4%)	
**Education**	445					
Less than high school	49	10.1%	18 (19.4%)	29 (12.8%)	2 (1.6%)	
High school/vocational training	203	47.2%	57 (61.3%)	108 (47.8%)	38 (30.2%)	
College and above	193	42.6%	18 (19.4%)	89 (39.4%)	86 (68.3%)	
**Household income**	444					<0.01
Less than $10,000	235	57.0%	57 (70.0%)	137 (63.8%)	41 (35.3%)	
$10,001 to $20,000	105	24.1%	19 (17.6%)	44 (20.2%)	42 (35.8%)	
>$20,000	82	18.9%	9 (12.4%)	35 (16.0%)	38 (28.9%)	
**Employment**	444					<0.01
Unemployed/student	253	56.7%	62 (66.8%)	130 (56.3%)	61 (49.4%)	
**Marital status**						0.31
Married/Living with a partner	148	29.7%	28 (26.7%)	70 (28.8%)	50 (33.7%)	

*Weighted.

**Interquartile range.

^+^Rao-Scott likelihood ratio chi-square test.

### Health literacy and health behaviors

The prevalence of many unhealthy behaviors was significantly associated with low health literacy (Table 2). Of those with inadequate health literacy, 19.8% reported currently smoking, compared to 15.8% of those with marginal health literacy and 8.8% of those with adequate health literacy (p < 0.01). Pregnant women with inadequate health literacy were also more likely to report not consistently keeping their appointments (16.8%), compared to those with marginal (6.9%) and adequate (2.1%) health literacy skills (p = 0.03). They were also less likely to have taken a vitamin every day during the thirty days prior to the baseline interview than those with marginal or adequate health literacy (45.1% versus 29.3% and 15.4%, respectively; p < 0.01). About 30% of postpartum women with less than adequate health literacy never breastfed their infant (compared to 13% if adequate; p < 0.01).

**Table 2:** Distribution of unhealthy behaviors in the text4baby program by health literacy categories, text4baby programs, two metro Atlanta WIC clinics, 2012

**Health behavior**	n = 445, Percent*	**Newest vital sign score**			p-value**
		**Limited = 0-1**	**Intermediate = 2-3**	**Adequate = 4-6**	
		**(n = 93)**	**(n = 226)**	**(n = 126)**	
Pregnant and postpartum samples, n, percent*					
*Do you currently smoke?*	60 (14.7%)	16 (19.8%)	33 (15.8%)	11 (8.8%)	<0.01
Some days/Every day					
*Do you have rules about smoking in your house?*					
No, people are allowed to smoke in the house	38 (8.6%)	7 (6.4%)	27 (13.1%)	4 (2.6%)	<0.01
*Have you had a flu shot in the past year?*					
No/Don’t know	319 (70.2%)	67 (71.1%)	153 (65.6%)	99 (77.4%)	0.01
*How much of the time have you felt downhearted and blue?*	24 (5.1%)	5 (5.4%)	14 (5.9%)	5 (3.4%)	0.38
Most/All of the time					
Pregnant sample only (n = 113), n, percent*					
*Have you had an alcoholic beverage in the last 30 days?*					
Yes/Don’t know	4 (3.0%)	0 (0%)	4 (6.0%)	0 (0%)	0.02
*How often do you exercise?*					
Less than 3 days a week (not on physical restriction)	53 (47.8%)	11 (45.4%)	29 (46.8%)	13 (52.4%)	0.76
*How often do you keep your appointments?*	9 (8.2%)	4 (16.8%)	4 (6.9%)	1 (2.1%)	0.03
Less than all of the time					
*How often do you take a vitamin?*					
Less than daily	31 (29.8%)	10 (45.1%)	17 (29.3%)	4 (15.4%)	<0.01
Postpartum sample only (n = 332), n, percent*					
*Are you currently breastfeeding or did you ever breast feed your child?*					
Never breastfed	86 (24.4%)	21 (27.9%)	51 (30.0%)	14 (12.6%)	<0.01
*How often do you put your child in a car seat?*					
Less than all of the time	6 (7.4%)	6 (10.8%)	14 (6.7%)	5 (6.1%)	0.33

*Weighted.

**Rao-Scott likelihood ratio chi-square test.

After controlling for income and education, only daily vitamin intake was significantly and consistently associated with lower health literacy across all categories (limited versus intermediate aOR 2.2, 95% CI 1.4, 3.6; limited versus adequate aOR 3.6, 95% CI 1.6, 8.5) (Table 3).

**Table 3:** Adjusted odds ratios for unhealthy behaviors in text4baby program by health literacy categories, text4baby programs, two metro Atlanta WIC clinics, 2012

**Predictor**		aOR (95% CI)*
	**Pregnant and postpartum samples**	
**Currently smokes**	Limited vs. Intermediate	1.2 (0.8, 1.7)
	Limited vs. Adequate	1.2 (0.8, 1.9)
**People are allowed to smoke in the house**	Limited vs. Intermediate	0.3 (0.2,0 .6)
	Limited vs. Adequate	1.2 (0.7, 2.2)
**Did not have a flu shot in the past year**	Limited vs. Intermediate	1.2 (0.9, 1.7)
	Limited vs. Adequate	0.6 (0.4,0 .9)
	**Pregnant sample only**	
**Does not always keep appointments**	Limited vs. Intermediate	1.0 (0.3, 4.1)
	Limited vs. Adequate	1.5 (0.4, 5.0)
**Does not take a daily vitamin**	Limited vs. Intermediate	2.2 (1.4, 3.6)
	Limited vs. Adequate	3.6 (1.6, 8.5)
	**Postpartum sample only**	
**Never breastfed**	Limited vs. Intermediate	0.7 (0.5,0 .9)
	Limited vs. Adequate	1.4 (1.1, 1.8)

*Adjusted for income and education.

### Health literacy and cell phone usage characteristics

Overall, an estimated 7.0% of the sample population shared cell phones, 24.6% had changed their cell phone number at least once in the six months prior to enrollment, and over two-thirds received 9 or more texts per day (Table 4). Sharing a cell phone was more than twice as common among those with the lowest health literacy scores (p < 0.01), but health literacy was not significantly associated with changing cell phone numbers or receiving more texts.

**Table 4:** Distribution of cell phone usage characteristics by health literacy categories, text4baby programs, two metro Atlanta WIC clinics, 2012

**Barrier**	n = 445 (Percent*)	**Newest vital sign score**	**Newest vital sign score**	**Newest vital sign score**	**Likelihood ratio**
		**Limited = 0-1**	**Intermediate = 2-3**	**Adequate = 4-6**	**Chi-square**
		n = 93 (Percent*)	n = 226 (Percent*)	n = 126 (Percent*)	**p-value**
**Shares a cell phone**	32 (7.0%)	12 (13.8%)	13 (5.3%)	7 (4.7%)	<0.01
**More than one phone number in the past six months**	107 (24.6%)	25 (27.8%)	57 (25.9%)	25 (19.8%)	0.15
**Average number of text messages received daily**					0.14
Less than 2	7 (5.8%)	8 (6.5%)	11 (4.6%)	8 (7.2%)	
2-8	125 (24.8%)	22 (20.5%)	61 (24.6%)	42 (28.6%)	
9 or more	293 (69.4%)	63 (73.0%)	154 (70.8%)	76 (64.1%)	

*Weighted.

For sharing a cell phone, NVS score remained predictive in the presence of all potential confounders, with those in the lowest health literacy category having 2.57 times the odds of sharing a cell phone compared to those with intermediate health literacy (95% CI 1.79, 3.69), and 1.67 times that of those with adequate health literacy (95% CI 1.06, 2.63) (Table 5). Health literacy was not independently associated with changing cell phone numbers or number of texts received daily. Younger women were significantly more likely to change phone numbers and receive more texts, and lower income was significantly associated with sharing a cell phone. Other factors were either not significantly associated with cell phone usage, or the results did not demonstrate a consistent trend.

**Table 5:** Multiple linear regression models of cell phone usage as a function of health literacy and other maternal characteristics, text4baby programs, two Metro Atlanta WIC clinics, 2012

		**Sharing a cell phone**	**Changed phone numbers at least once in the previous six months**	**Receiving nine or more messages a day**
**Predictor**		**OR (95% CI)**	**OR (95% CI)**	**OR (95% CI)**
**NVS score**	Limited vs. Intermediate	2.6 (1.8, 3.7)	1.1 (0.8, 1.4)	1.2 (0.9, 1.6)
	Limited vs. Adequate	1.7 (1.1, 2.6)	1.4 (0.9, 2.0)	1.38 (1.0, 1.9)
**Maternal age**	(18–22) vs. (22–28)	1.3 (0.8, 2.1)	1.5 (1.1, 2.0)	1.9 (1.4, 2.6)
	(18–22) vs. (28–46)	0.4 (0.30, 0.60)	1.8 (1.2, 2.6)	5.1 (3.8, 6.8)
**Income**	(<$10,000) versus ($10,000-$20,000)	2.2 (1.2, 4.0)	1.2 (0.9, 1.7)	1.4 (1.1, 1.9)
	(<$10,000) versus (>$20,000)	4.2 (1.5,12.2)	1.2 (0.9, 1.7)	0.6 (0.5, 0.8)
**Education**	<HS versus HS/vocational training	0.9 (0.5, 1.7)	0.9 (0.6, 1.2)	1.2 (0.7, 2.0)
	<HS versus some college or above	1.7 (0.9, 3.3)	1.8 (1.2, 2.6)	1.2 (0.7, 2.0)
**Employment**	Employed versus Unemployed/Student	1.0 (0.7, 1.5)	0.7 (0.6, 0.9)	0.9 (0.7, 1.1)

## Discussion

In this study population, lower health literacy was significantly associated with a variety of unhealthy behaviors that are known to have a negative impact on maternal and infant health. This is consistent with several studies that have found a similar association with low health literacy and certain unhealthy behaviors, including smoking and not receiving an influenza vaccine [11]. Fewer studies, however, have looked directly at the target population of text4baby, pregnant and postpartum women. Importantly, daily prenatal vitamin intake was mediated by health literacy in our sample even after controlling for confounders, making this an important target for future mhealth programs aimed at lower health literacy levels.

Given the higher prevalence of unhealthy behaviors amongst the lowest health literacy groups, it is important that future analyses of text4baby examine the relative impact of the program at different literacy levels. Though the developers have written messages that are meant to accommodate lower health literacy levels, the messages may need to be simplified further: limited health literacy on the NVS corresponds to less than a sixth grade reading level, the level at which text4baby messages are written. Supplemental information delivery, which several studies have found to be effective, may be incorporated in the future, especially using smart phone platforms. These supplemental delivery methods include using videos, icons, and verbal narratives [11].

This study is also one of the first to examine directly how people enrolled in an mhealth program use their cell phones, and how these patterns of usage are related to health literacy and demographic variables. Our analysis shows that those with low health literacy are more likely to share a cell phone, and this relationship remained significant after adjusting for age, sex, income, education, and employment status. Those in the lowest income category were also more likely to share a cell phone. The youngest group (ages 18–22) was the most likely to have changed their cell phone number at least once in the previous 6 months, as were the unemployed. The youngest participants were also the most likely to receive nine or more text messages a day. These findings are largely consistent with national surveys of text messaging. Our findings are also supported by data that the youngest Americans are more likely to share a cell phone [6]. Though we did not find data on cell phone number instability, low income populations are more likely to use prepaid cell phone cards, and therefore more likely to experience service disruptions [5]. Use of prepaid plans that do not require reading and signing a complicated contract and are also less expensive may be more common among women with lower health literacy. So far, however, this relationship has not been explored directly.

To determine the effectiveness of text4baby, researchers will need to determine if rates of knowledge acquisition and behavior change differ depending on health literacy skills. Text4baby continues to be promising in this population given that it incorporates a few core features of effective communication with low health literacy populations, namely presenting important information by itself and using limited numeracy in messages [11]. It is possible that text message may not be the most effective medium to reach those with low health literacy, or that supplemental learning aides will be necessary to effect change in this population. The Agency for Healthcare Research and Quality, for instance, found in their systematic review that those with low health literacy benefit from visual aides and videos. Given the rapid expansion of smartphones, which are now available on many prepaid plans, text4baby could explore the advantages and use of these expanded platforms.

Several mhealth interventions have successfully improved health behaviors known to impact maternal and infant health [10-14]. Mhealth programs have rarely, however, examined directly the ways that participants use their cell phones, or the ways that these usage patterns may affect the design, measurement, or retention of these programs. Drop-off is a particularly important problem for our study and larger mhelath studies in general. One pilot study of text message reminders for parents in a low-income urban clinic found that 19 of 48 participants changed their number at seven-month contact, and were thus lost-to-follow-up [19]. Determining what leads to successful retention in these programs is essential to designing an intervention that can be evaluated and scaled up beyond a pilot study. As of yet, very little data is available on what leads to drop-off and how it might be prevented.

There are several limitations of our research. Health behaviors were self-reported, and therefore may not represent the true behavior of the baseline population, especially for socially undesirable behaviors. For instance, only four participants indicated that they drank during pregnancy. Secondly, there were few events, and therefore not enough events to control for multiple confounders. We also did not ask participants for more information about their cell phone usage patterns, particularly whether they used prepaid cell phone cards or had long-term service plans. In large-scale surveys, these plans are more common among low-income populations, and therefore were likely common in our study sample. This may be an important factor in the usage patterns we found. Fourth, the relationship between health literacy and other predictors with these cell phone usage characteristics may have been underestimated in this population, as it was primarily a low literacy population and fairly homogenous with respect to demographic variables. We also do not have data on how long participants continued to receive text4baby messages after enrollment, and therefore cannot infer how these different usage patterns would affect retention or receipt of messages. Finally, our use of women from two urban clinics may not be generalizable to the whole population, and cell phone usage may vary by region.

Despite the noted limitations, this study provides insight to the ideal design of mhealth programs, particularly for low health literacy and low resource populations in urban centers. As preliminary data and surveys indicate that the ways that people use their cell phones is not uniform, this is particularly important for programs like text4baby that are aimed at large and diverse populations. In addition, the study population is similar to the ideal target population of the national text4baby program, with participants having significant health burdens and low health literacy.

One implication of this study is that mhealth participants should be asked about how they use their cell phone. If targeting those with low health literacy or other groups who may be more likely to share a cell phone, designers of mhealth programs should consider how they will determine that the intended recipient actually read the message, and how they will ensure privacy of the participant. Programs could build in ways to determine that the intended recipient had read the message by: using the name of the recipient, having them text back, providing a free cell phone to recipient, or sending password-protected messages. In promoting retention, program designers should consider if their participants are likely to change their numbers often, especially if their target group is young, unemployed, or has less than some college education. The appropriateness of text messaging as a means of targeting low socio-economic and health literate populations is reaffirmed by our study, as the majority of participants receive more than nine messages a day. This, however, means that text4baby and similar mhealth programs must rise above the noise of other messages that enrollees receive in a given day.

Future studies of text4baby should identify opportunities to determine that the intended recipient has received the message. They should also measure retention directly to determine what infrastructural barriers may lead to drop-off. Finally, participants should be asked directly about how they use their cell phones and ways that text4baby could more effectively address the needs of its target population. Addressing these infrastructural issues is an important step in refining the design of these programs and measuring their impact.

## Conclusion

Health promotion through text messaging is a promising avenue to target maternal and infant health given the high prevalence of text messaging among women who are young and have lower health literacy. The ways in which low health literacy groups use their cell phones should affect the design of text4baby and similar programs to ensure success. Participants in these programs should be asked about the ways in which they use their cell phones in order to ensure receipt of messages, privacy amongst groups more likely to share phones, and penetrance of the messages amongst high volume texters.

## Competing interests

The authors state that they have no financial or competing interests.

## Authors’ contributions

EP performed the literature search, created the models, and drafted the manuscript. JG was the Principal Investigator of the main study and oversaw all aspects of the study design and implementation. She also reviewed and commented on several versions of the manuscript. LE supervised the study design, data management and statistical analysis, and drafting of the manuscript. RP was a co-investigator of the main study and provided extensive feedback on the survey instruments and drafts of the manuscript. All authors read and approved the final manuscript.
